# Emotions and virality: Social transmission of political messages on Twitter

**DOI:** 10.3389/fpsyg.2022.931921

**Published:** 2022-11-11

**Authors:** Niklas Pivecka, Roja Alexandra Ratzinger, Arnd Florack

**Affiliations:** ^1^Department of Occupational, Economic and Social Psychology, Faculty of Psychology, University of Vienna, Vienna, Austria; ^2^Institute of Leadership and Change Management, Johannes Kepler University Linz, Linz, Austria

**Keywords:** social networks, emotions, Twitter, virality, political communication, information diffusion, social transmission

## Abstract

Drawing on previous literature that valence and arousal constitute the fundamental properties of emotions and that emotional content is a determinant of social transmission, this study examines the role of valence and arousal in the social transmission of politicians’ messages on Twitter. For over 3,000 tweets from five Austrian party leaders, the discrete emotion that the message intended to elicit in its recipients was captured by human coders and then classified on its valence (positive or negative) and arousal (low or high). We examined the effects of valence and arousal on the retweet probability of messages. Results indicate that tweets eliciting a negative (vs. positive) valence decreased retweet probability, whereas tweets eliciting a high (vs. low) arousal increased retweet probability. The present research replicates previous findings that arousal constitutes a determinant of social transmission but extends this mechanism to the realm of political communication on Twitter. Moreover, in contrast to the frequently mentioned negativity bias, positive emotions increased the likelihood of a message being shared in this study.

## Introduction

The short message service Twitter is frequently used by politicians to attract voters and communicate and interact with supporters. It is an important tool for influencing public opinion and discourse (Jungherr, 2016; Getachew and Beshah, 2019). Moreover, a high interactivity of politicians on Twitter can lead to more positive evaluations and heightened social presence (Lee and Shin, 2012). Users can follow a certain politician to be directly exposed to his or her messages, but also can retransmit the message to their followers (Suh et al., 2010). Thus, politicians can expand their reach on Twitter by composing messages more likely to be shared by their followers.

Messages containing useful information are shared more often than messages without any utility for the user and its followers (Rudat and Buder, 2015). However, it is not only the informational value of a message that drives social transmission (Cappella et al., 2014). Especially the emotional content of a message has been identified as a relevant determinant of social transmission (e.g., Berger and Milkman, 2012; Stieglitz and Dang-Xuan, 2013; Tellis et al., 2019). This study takes a closer look at which attributes of a message foster social transmission in the realm of political communication on Twitter. In particular, we focus on how emotional content influences the virality of politicians’ tweets. We do so by having human coders indicate for the tweets of five Austrian politicians which emotion the tweets elicit in the recipients. We then classify the valence and the arousal of the emotions based on the well-established affective circumplex model (Russell, 1980) and analyze what kind of tweets get retweeted most. To the best of our knowledge, this is the first study to analyze the valence and arousal of distinct emotions in a political context on Twitter.

Our study contributes to the literature in two aspects: First, we replicate and extend existing findings regarding the relationship between arousal and social transmission. While using a similar approach as Berger and Milkman (2012), we focus on implications for politicians instead of advertisers and marketers and use Twitter data instead of newspaper articles. Extending results to the realm of political communication on Twitter is highly relevant because Twitter is an increasingly important communication tool for politicians to influence voters. Difficulties in replicating study results in different contexts have led to extensive discussions in multiple disciplines and underline the importance of this study (Klein et al., 2014; Schooler, 2014). Moreover, extensive research on Twitter communications investigating the emotional content of messages mainly relies on sentiment analysis programs (e.g., Stieglitz and Dang-Xuan, 2013) and fails to capture discrete emotions and their associated valence and arousal. Second, we explore the role of valence in social transmission. While some studies propose a negativity bias in social transmission (Bebbington et al., 2017), different studies found that messages with positive emotions are more likely to be shared (Ferrara and Yang, 2015). Thus, our study helps to resolve inconsistencies in previous research. In the following, we will review literature about the dimensionality of emotions and emotional antecedents of sharing behavior.

## Literature review

Emotions can be divided into different components, with valence (hedonicity) and arousal (activation) referred to as the fundamental dimensions of emotions (Bliss-Moreau et al., 2020). The affective circumplex model relies on both dimensions to conceptualize an emotion as a single point in a two-dimensional space (Russell, 1980; Barrett and Russell, 1998). Importantly, these dimensions are largely independent, meaning that emotions with the same valence can still differ in the arousal they induce (Russell, 1980). For example, anger and anxiety are negative emotions inducing a high arousal, whereas sadness elicits low arousal. Joy, amusement, and deference (or awe) are positive emotions eliciting high arousal, whereas gratitude and contentment are associated with low arousal (Russell, 1980; Bliss-Moreau et al., 2020).

In an analysis of nearly 7,000 The New York Times articles, emotional arousal was identified as predictor of social transmission, independent of valence (Berger and Milkman, 2012). Both, articles which evoked a positive and negative valence were more likely to go viral if they also evoked high instead of low arousal. As arousal is connected to an excitatory state of mobilization and general readiness to respond (Heilman, 1997), being exposed to messages that elicit high arousal could lead to an urge of action, which in turn could be satisfied by sharing these messages with other people (Berger and Milkman, 2012).

While findings regarding arousal as a determinant of social transmission are robust (Berger, 2011; Berger and Milkman, 2012), literature provides contradictory findings regarding the valence of a message (Eckler and Bolls, 2011; Bebbington et al., 2017). On the one hand, positive emotions might foster social transmission as sharing positive information is connected to status-seeking and self-enhancement (Wojnicki and Godes, 2008). On the other hand, negative information may possess a saliency advantage (Baumeister et al., 2001; Bebbington et al., 2017). Hence, some authors suggest that a negativity bias is prevalent for information selection (Meffert et al., 2006; Soroka and McAdams, 2015; Knobloch-Westerwick et al., 2020), whereas a positivity bias is prevalent for information transmission (Eckler and Bolls, 2011).

Research has increasingly focused on how emotions shape information diffusion on Twitter (Chawla and Mehrotra, 2021). Similar to the findings regarding social transmission in general, some authors report that tweets eliciting positive emotions are shared more often (e.g., Ferrara and Yang, 2015), whereas others suggest an advantage for negative emotions in certain contexts (Brady et al., 2017). However, distinct emotions in tweets are rarely examined (e.g., So et al., 2016; Glasgow et al., 2017; Chawla and Mehrotra, 2021). In a recent study, Wang and Lee (2020) found that cancer tweets evoking anger are shared more often than tweets evoking sadness, supporting that high arousal emotions foster social transmission compared to low arousal emotions.

In a political context, the relationship between discrete emotions and information diffusion on Twitter has not been investigated so far. In one study, the weighted frequency of negative and positive words in 165,000 tweets regarding federal state elections in Germany predicted social transmission (Stieglitz and Dang-Xuan, 2013). Tweets with many positive and negative words were more likely to be retweeted and more quickly retweeted. The authors interpreted the absolute frequencies of positive and negative words as the emotional intensity of a tweet. The words were classified as positive or negative using a sentiment analysis program. Similar sentiment analysis tools are frequently used to investigate the influence of emotional content on social networks (e.g., Vargas et al., 2021). Furthermore, a wide range of machine learning and deep learning methods exist to study predictors of sharing behavior (e.g., Zhang et al., 2016; Wang and Yang, 2021). Investigated predictors include user characteristics such as personality and activity (e.g., Fu et al., 2022; Sharma and Gupta, 2022) but also emotional content of tweets (e.g., Firdaus et al., 2021; Rodrigues et al., 2022). Recently, researchers also applied a Bayesian modelling approach to predict retweet probability as function of the content and the emotional features of a tweet (Casarin et al., 2021). However, these methods usually do not measure intended or elicited emotional arousal or discrete emotions.

Whereas Stieglitz and Dang-Xuan (2013) measured the frequency of positive and negative words in a tweet, our study attempts to identify the most likely intended discrete emotion of a message and its associated valence and arousal. Single words of a message can vary in their emotional sentiment, but short messages, such as those written on Twitter, should, in most cases, only transport one discrete emotion. Furthermore, messages can elicit emotions when no emotional word is present but the context implies a discrete emotion. For example, in his last tweet before being banned from the platform, Donald Trump wrote to his followers that he “will not be going to the Inauguration [of Joe Biden as president of the United States] on January 20th” (Twitter Blog, 2021). The tweet does not contain any emotional words but clearly intends to elicit a discrete emotion in its recipients (most likely anger). Hence, this study aims to capture the discrete emotion transported in a tweet using human coders.

In addition, no distinction between politicians and other users of Twitter has been made. Yet, as politicians use Twitter differently and more strategically than other users (Stier et al., 2018), it is reasonable to assume that they also compose their messages more strategically. For instance, Colombian politicians were able to identify and use combinations of sensitive words during political campaigns that increased retweet probability on Twitter (Casarin et al., 2021). Thus, strategic use of Twitter by politicians can increase retweet probability, which in turn influences election results (e.g., Correa and Camargo, 2017). Our study investigates which discrete emotions politicians intend to evoke in recipients of their messages and whether the effect of the intended discrete emotions on the sharing behavior of the recipients is in line with effects observed in previous research.

We predicted that messages (tweets) by politicians which intend to evoke high arousal will be shared (retweeted) more often than messages which intend to evoke low arousal. Moreover, we took the reasoning into account that a negativity bias is prevalent for information selection and a positivity bias for information transmission. Hence, we expected that messages evoking positive emotions should be shared more often than messages evoking negative emotions.

## Materials and methods

### Data collection

The tweets from the five party leaders of the Austrian Parliament in 2018 were collected using Twitter’s Rest API. The party leaders at that time were Sebastian Kurz (@sebastiankurz) for the People’s Party (OeVP), Heinz-Christian Strache (@HCStracheFP) for the Freedom Party (FPOe), Christian Kern (@KernChri) for the Social Democrats (SPOe), Matthias Strolz (@matstrolz) for the Austrian liberals (NEOS) and Peter Pilz (@Peter_Pilz) for the Peter Pilz List (a founding member of the Austrian Green Party). The original composition of tweets was within a period between 06.09.2006 and 30.09.2018. Retweets, replies, non-German tweets, redundant tweets, and tweets shorter than five words were excluded, which resulted in a data set of 5,899 tweets. Emoticons were excluded from all tweets because the interplay between written text and emoticons in ambiguous messages is complex and behaves differently for positive and negative messages (Alduante et al., 2018) In addition, it is a problem that emoticons can appear differently on different devices and are not shown on some devices. For every tweet, we used information about its retweet count as criterium for social transmission on Twitter.

### Data coding

One-hundred and eighty-eight human coders (*M_Age_* = 21.37, *SD_Age_* = 2.57; 73% female) were recruited at a large Austrian University to classify the extent to which a tweet intended to evoke the following eight emotions: anger, anxiety, deference, joy, amusement, sadness, gratitude, and contentment. Following Berger and Milkman (2012), we chose anger, anxiety, sadness, and deference (or awe) because these emotions significantly predicted the likelihood of a news article being shared. Joy, amusement, gratitude, and contentment were added because these emotions cover the area of positive emotions on Twitter in addition to deference (e.g., So et al., 2016; Glasgow et al., 2017; Chawla and Mehrotra, 2021). We also selected these emotions to ensure we measured positive and negative emotions with high and low arousal.

Every coder was randomly assigned 30 tweets out of the data set. After the presentation of the tweet, the coders rated all eight emotions on whether the tweet intended to evoke the specific emotion using a 5-point Likert scale (“not at all” to “very much”). Coders rated the tweets without information about the author of the tweet or retweet count. We chose this approach because users on Twitter perceive tweets at different times and with different retweet counts. Also, we did not name the author to reduce the influence of preexisting attitudes towards the politicians. However, coders indicated that they understood the topic and context for the vast majority of the tweets (i.e., 92% of the tweets). Coders were explicitly instructed to rate tweets as objectively as possible on the intended effect on the original recipients. In total, coders rated 3,404 tweets. Ratings for the same tweet were not averaged, but we treated every rating as single observation. We decided to use a large sample of human coders (instead of just a few) for three reasons. First, using multiple human coders allowed us to generate ratings for a large number of tweets. Second, multiple coders reduced method biases due to response tendencies. Third, we were interested in the ratings of different coders because emotion recognition accuracy is higher aggregated over many peoples’ judgments (Yi et al., 2012; Sager et al., 2019). In order to avoid ambiguity regarding the valence and arousal of a tweet, we reduced tweets to their highest-rated emotion. Thus, tweets were excluded when one coder assigned two or more emotions the highest value, leaving 3,214 rated tweets for the final data analysis.

**Figure 1:**
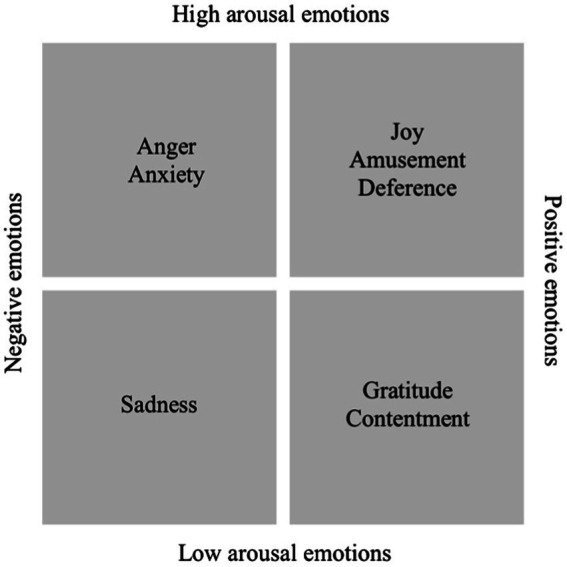
Classification of the emotions studied based on their valence and arousal. The horizontal axis depicts the valence dimension, and the vertical axis is the arousal dimension.

We classified the emotions as possessing a positive (joy, amusement, gratitude, deference, contentment) or negative (anger, anxiety, sadness) valence and a low (gratitude, sadness, contentment) or high (anger, anxiety, joy, amusement, deference) arousal (see Figure 1). As a result, every tweet obtained a dichotomous manifestation of its evoked valence and arousal, which served as predictors for social transmission on Twitter. Classifications are based on the affective circumplex model (Russell, 1980). All (or very similar) emotions were classified by Russell (1980) or later studies relying on the affective circumplex model (Bliss-Moreau et al., 2020).

## Results

### Preliminary analysis

Anger was by far the most classified emotion. Anger was the highest rated emotion in 47% of the analyzed tweets. The second highest rated emotion was deference (11%). Consequently, 56% of all tweets evoked negative emotions and 82% high arousal. The number of total tweet counts per emotion with the average retweet count per emotion can be found in Table 1.

**Table 1:** Studied emotions with retweet count per emotion.

Emotions					Retweet Count	
	*n*	*%*	Valence	Arousal	*M*	*SD*
Anger	1,508	46.91	Negative	High	26.94	53.05
Deference	341	10.61	Positive	High	27.62	60.66
Contentment	322	10.02	Positive	Low	24.06	36.51
Joy	315	9.80	Positive	High	26.49	44.29
Amusement	292	9.09	Positive	High	39.07	89.85
Anxiety	180	5.60	Negative	High	28.51	38.21
Gratitude	156	4.85	Positive	Low	23.58	35.75
Sadness	100	3.11	Negative	Low	27.87	40.67
Total	3,214	100	–	–	27.74	54.53

### Statistical approach

A Generalized Linear Mixed Model (GLMM) was applied to estimate the relationship between valence and arousal of a tweet and its retweet probability. The dependent variable, retweet count, constitutes true-event data (no negative values and integers only) with a larger standard deviation than its mean (*M* = 27.74, *SD* = 54.53). Thus, we applied a negative binomial distribution to the model using a log-link. The negative binomial regression model was used in previous studies to analyze retweet counts on Twitter (e.g., Stieglitz and Dang-Xuan, 2013).

The valence and arousal of a tweet were included as fixed effects. Valence and arousal are bipolar variables with positive valence and low arousal as the reference category for the respective variable. In addition, fixed effects were calculated for the number of hashtags in a tweet, whether tweets have a link to an external source, and time of day, to control for these variables. The number of hashtags and time of day yielded significant coefficients. Tweets with more hashtags (*p* < 0.001) and tweets composed in the evening (vs. night; *p* = 0.029) increase retweet probability. However, the control variables did not affect the results for valence and arousal, so only the results for valence and arousal are reported in detail. All estimates of the fixed effects (including control variables) with corresponding confidence intervals on the response scale and variances in random intercepts can be found in Table 2.

**Table 2:** Results of a generalized linear mixed model with retweet count as dependent variable.

	Retweet Count		
	IRR	CI	*p*
*Fixed Effects* (Intercept)	16.35	9.56–27.97	<0.001
Arousal [high]	1.17	1.05–1.31	0.004
Valence [negative]	0.88	0.81–0.96	0.003
Number of hashtags	1.14	1.10–1.18	<0.001
URL-Link [yes]	1.07	0.97–1.19	0.170
Daytime			
Morning (8 am – 2 pm)	1.07	0.95–1.21	0.267
Afternoon (2 pm – 7 pm)	0.96	0.84–1.09	0.501
Evening (7 pm – 10 pm)	1.20	1.02–1.42	0.029
*Random Effects*			
σ^2^	0.76		
τ_00 coder_	0.08		
τ_00 politician_	0.34		
ICC	0.36		
N _coder_	188		
N _politician_	5		
Observations	3,214		
Marginal *R*^2^/Conditional *R*^2^	0.032/0.376		

IRR, Incidence rate ratio; CI, Confidence interval; σ^2^, residual variance; τ_00_, random intercept variances; ICC, Intraclass correlation coefficient. Arousal, valence, URL-Link, and daytime are dummy-coded variables with the following reference categories: arousal, “low arousal”; valence, “positive valence”; URL-Link, “no URL-Link”; Daytime, “night (10 pm – 8 am).”

In order to account for variance in retweet count between politicians (due to follower numbers and tweet activity, among others), random intercepts were permitted for the politicians who composed the tweets. Random intercepts were also applied for the human coders because mean retweet count might differ between coders due to the random assignment of tweets. Mean and standard deviation for retweet count and the number of tweets by politician, valence, and arousal are summarized in the Supplementary Table S1. The model with both random effects achieved a better fit than models with only the politician (𝜒^2^(1, *N* = 3,214) = 62.94, *p* < 0.001) or the coder (𝜒^2^(1, *N* = 3,214) = 498.06, *p* < 0.001) as random effect. All analyses for estimating model parameters were conducted using the *lme4* package (version 1.1–26; Bates et al., 2015) in R (R Core Team, 2020).

### Main analysis

Valence (*b* = −0.13, *z* = −2.97, *p* = 0.003, 95% CI [−0.21, −0.04]) and arousal (*b* = 0.16, *z* = 2.85, *p* = 0.004, 95% CI [0.05, 0.27]) of a tweet significantly predicted its retweet probability. To enhance interpretability, estimated marginals means (i.e., least-square means) and standard errors on the response scale are reported, and we back-transformed coefficients to rate ratios by calculating the exponentiated coefficients. Rate ratios can be used to represent the percentage change in the dependent variable as the result of the independent variables. Values over 1 indicate a percentage increase, whereas values under 1 indicate a percentage decrease compared to the reference category.

Tweets with a high arousal (*M* = 22.16, *SE* = 5.86) were retweeted more often than tweets with a low arousal (*M* = 18.92, *SE* = 5.07) and tweets eliciting emotions with negative valence (*M* = 19.21, *SE* = 5.11) were retweeted less often than tweets eliciting emotions with positive valence (*M* = 21.82, *SE* = 5.79). The rate ratios illustrate that high arousal (exp(*b*) = 1.17, CI [1.05, 1.31]) increases the retweet probability by 17% compared to low arousal. On the other hand, a negative (vs. positive) valence (exp(*b*) = 0.88, CI [0.81, 0.96]) reduces retweet probability by 12%. Importantly, the applied model ensures that the statistical effects of arousal and valence on social transmission cannot be explained by the politician.

## Discussion

Our analysis of more than 3,000 tweets composed by Austrian politicians supports the notion that the emotional content of a message is a driving force for social transmission in the online world. In more detail, tweets that intended to evoke an emotion with high arousal were retweeted more often than tweets intended to evoke an emotion with low arousal. Furthermore, tweets containing positive emotions were more likely to be retweeted than tweets evoking negative emotions.

Our study highlights the importance of the arousal and valence dimension from the affective circumplex model for social transmission (Russell, 1980). The finding that emotional arousal is a relevant factor for the social transmission of politicians’ messages on Twitter fits observations regarding the relationship between arousal and social transmission in other fields. In particular, our results support previous findings that the activating potential of emotional arousal transfers to sharing behavior (Berger, 2011; Berger and Milkman, 2012).

Research has provided mixed results about whether the effect of valence on social transmission reflects a positivity (Eckler and Bolls, 2011; Ferrara and Yang, 2015) or negativity bias (Bebbington et al., 2017; de León and Trilling, 2021). In the present study, we find support for a positivity bias in the social transmission of politicians’ messages. One explanation might be that people prefer to be the messenger of positive news (Wojnicki and Godes, 2008) and are reluctant to share negative tweets even if the negative tweets elicit a state of readiness to act.

The present study adds to the existing literature by pointing out that a relationship between discrete emotions and social transmission of information also exists in a political context on Twitter. So far, arousal has been identified as predictor for social transmission in a database including all kinds of topics and experimental designs, with implications for advertisers and marketers (Berger and Milkman, 2012). To the best of our knowledge, the present study is the first that identifies emotional arousal as relevant attribute for social transmission in political communication on social network sites. Furthermore, by using human coders and focusing on discrete emotions, we extend previous literature that has studied the effect of political content on Twitter using sentiment analysis tools (Stieglitz and Dang-Xuan, 2013). Research concerning emotional content on Twitter usually relies on automatic coding procedures (e.g., Matalon et al., 2021). Hence, we validate previous research that has investigated the effects of emotional content on Twitter and extend it, as sentiment analysis tools rarely capture distinct emotions and are often not available in languages other than English.

An important limitation of the present study is that the applied approach did not allow to compute the intercoder reliability. An alternative approach would have been to use few coders for all tweets. However, we deliberately refrained from using very few coders to assess all tweets, because the number of tweets was very high. Using few coders for many tweets has two main disadvantages. First, coding many tweets is likely to deplete the coders and affect the ability to identify the intended emotions in the tweets. Second, using few coders for all tweets could increase the likelihood that the assessments are biased by individual perceptions of single coders. By contrast, the applied approach has the advantage that perceptions of many different individuals are measured – very much like what is the case for Twitter messages in reality. Hence, our approach represents the idea that the coded stimuli as well as the coders are sampled (Wells and Windschitl, 1999).

In addition, it is important to note that we did not assess arousal and valence directly. Direct measures of arousal and valence are available (Bradley and Lang, 1994). We asked participants to rate discrete emotions instead of arousal and valence to facilitate the coding. Distinct emotions are more easily recognizable for human raters than the extraction of arousal and valence from an emotion (e.g., in the recognition of facial expressions; Young et al., 1997). Hence, we categorized the discrete emotions rather than asking participants to rate valence and arousal. We did not analyze separate discrete emotions because we had no hypotheses about the single emotions and our sample size was too small to allow an exploration of such effects. However, it has to be taken into account that we have selected emotions we regarded as important for the tweets used in political communication. We have not measured all kinds of emotions and the results are limited to the emotions we have assessed.

For political communication on Twitter, the COVID-19 pandemic provides an intriguing scenario to examine the generalizability of our findings in different contexts. Research on sharing behavior of COVID-19 news mostly focuses on the transmission of negative emotions and misinformation (e.g., Lăzăroiu and Adams, 2020; Lăzăroiu et al., 2020; Kim and Florack, 2021; Vargas et al., 2021; Kim et al., 2022). Only a few studies have investigated how positive emotions like hope or optimism are shared during the COVID-19 pandemic (Nanath and Joy, 2021). Our study results would suggest that tweets eliciting positive emotions like hope or optimism should be shared more often than tweets eliciting negative emotions with low arousal like sadness.

## Conclusion

Politicians increasingly use Twitter to influence voters and shape public opinion. An important instrument to reach larger audiences is encouraging followers to share messages. In this paper, we show that tweets of Austrian politicians are more likely to be shared if they transport positive emotions or emotions accompanied by high arousal.

Interestingly, most analyzed tweets were classified as evoking emotions with high arousal. A possible explanation for this finding might be that politicians are implicitly or explicitly aware of the potential of emotional arousal for social transmission and strategically compose messages which evoke high arousal. However, our results suggest that the frequently observed intention to elicit negative emotions with tweets may be less optimal for increasing sharing. According to our results, tweets that elicit positive emotions with high arousal might be those that recipients frequently share.

The results of our study imply a positivity bias for information transmission, whereas literature reports a negativity bias for information selection (e.g., Knobloch-Westerwick et al., 2020). This reverse relationship could have great relevance for politicians trying to maximize their output on social networks. However, our study cannot draw any conclusions here, as we only investigated information transmission. In addition, we analyzed a particular context, namely Austrian politics. Future research should extend our findings to different political contexts (e.g., topics, countries) and investigate whether different mechanisms apply for information selection and transmission.

## Data availability statement

The dataset generated during the current study is not publicly available due to Twitter’s Developer Agreement but is available from the corresponding author on reasonable request.

## Ethics statement

Ethical review and approval was not required for the study on human participants in accordance with the local legislation and institutional requirements. The patients/participants provided their written informed consent to participate in this study.

## Author contributions

AF and RR conceptualized and planned the study. RR prepared and collected data for the study. NP and RR were involved in analyzing and interpreting the data. NP and AF wrote the first draft of the manuscript. All authors contributed to the article and approved the submitted version.

## Conflict of interest

The authors declare that the research was conducted in the absence of any commercial or financial relationships that could be construed as a potential conflict of interest.

## Publisher’s note

All claims expressed in this article are solely those of the authors and do not necessarily represent those of their affiliated organizations, or those of the publisher, the editors and the reviewers. Any product that may be evaluated in this article, or claim that may be made by its manufacturer, is not guaranteed or endorsed by the publisher.

## Supplementary material

The Supplementary material for this article can be found online at: https://www.frontiersin.org/articles/10.3389/fpsyg.2022.931921/full#supplementary-material
