# Supplementary material for: Emotions and virality: Social transmission of political messages on Twitter
Source: Front Psychol. 2022 Nov 11;13:931921. doi: 10.3389/fpsyg.2022.931921 (PMC9692101; doi:10.3389/fpsyg.2022.931921)
Supplement: Supplementary file 1 [file Table_1.DOCX]

Supplementary Material

# Tables

**Table S1**

*Descriptive statistics for retweet count by politician, valence and arousal*

| Emotion | | Strache | | |  | Kurz | | |  | Kern | | |  | Strolz | | |  | Pilz | | |  | Total | | |
| --- | --- | --- | --- | --- | --- | --- | --- | --- | --- | --- | --- | --- | --- | --- | --- | --- | --- | --- | --- | --- | --- | --- | --- | --- |
|  | | *n* | *M* | *SD* |  | *n* | *M* | *SD* |  | *n* | *M* | *SD* |  | *n* | *M* | *SD* |  | *n* | *M* | *SD* |  | *n* | *M* | *SD* |
| Valence | |  |  |  |  |  |  |  |  |  |  |  |  |  |  |  |  |  |  |  |  |  |  |  |
|  | Positive | 638 | 22.64 | 43.36 |  | 319 | 54.04 | 96.11 |  | 119 | 37.29 | 44.29 |  | 139 | 15.16 | 33.24 |  | 211 | 11.23 | 13.93 |  | 1426 | 28.47 | 58.56 |
|  | Negative | 766 | 20.02 | 32.77 |  | 427 | 49.65 | 82.34 |  | 183 | 40.05 | 49.18 |  | 178 | 11.73 | 23.68 |  | 234 | 11.10 | 11.22 |  | 1788 | 27.15 | 51.10 |
| Arousal | |  |  |  |  |  |  |  |  |  |  |  |  |  |  |  |  |  |  |  |  |  |  |  |
|  | Low | 256 | 20.29 | 30.76 |  | 127 | 46.11 | 53.23 |  | 50 | 33.46 | 37.68 |  | 54 | 7.93 | 8.82 |  | 91 | 11.66 | 15.44 |  | 578 | 24.59 | 37.03 |
|  | High | 1148 | 21.41 | 39.40 |  | 619 | 52.63 | 94.06 |  | 252 | 40.06 | 48.93 |  | 263 | 14.32 | 30.69 |  | 354 | 11.04 | 11.73 |  | 2636 | 28.43 | 57.65 |
| Total^a^ | | 1404 (1044) | 21.21 (20.74) | 37.96 (37.63) |  | 746  (573) | 51.52  (51.15) | 88.46 (90.20) |  | 302 (232) | 38.97 (38.95) | 47.26 (45.44) |  | 317 (226) | 13.23 (13.02) | 28.28 (28.54) |  | 445 (334) | 11.16 (11.51) | 12.56 (12.24) |  | 3214  (2409) | 27.74  (27.72) | 54.53  (55.33) |

*Note.* ^a^ Due to the random assignment of tweets, some tweets were assigned to more than one human coder and thus, can have more than one rating. Number of unique tweets by politician are shown in parentheses.
